# Advancing the taxonomy of *Sclerotinia* (Helotiales, Sclerotiniaceae): a review and recommendations for an important plant-pathogenic genus

**DOI:** 10.3897/imafungus.17.175737

**Published:** 2026-01-22

**Authors:** Chanel Thomas, P. Markus Wilken, Martin P. A. Coetzee, Cobus M. Visagie

**Affiliations:** 1 Department of Biochemistry, Genetics and Microbiology, Forestry and Agricultural Biotechnology Institute (FABI), University of Pretoria, Pretoria, South Africa University of Pretoria Pretoria South Africa

**Keywords:** Phylogenetics, *Sclerotinia minor*, *Sclerotinia sclerotiorum*, *Sclerotinia trifoliorum*, systematics

## Abstract

*Sclerotinia* is a fungal genus of significant agricultural and scientific importance, as it includes multiple plant pathogens and provides an informative case study for mechanisms of host generalism. However, the taxonomy of this group remains unsettled, which hinders research on these pathogens. The last monographic treatment of *Sclerotinia* was published more than 40 years ago and was centered on the morphological data available at that time. Here, we examine that revision alongside other pivotal publications to trace the taxonomic history of *Sclerotinia* and to evaluate the morphological traits used to identify *Sclerotinia* species. We also briefly assess the composition of genera in the family *Sclerotiniaceae*, emphasising the need for a modern taxonomic investigation of the broader group. Thirteen new *Sclerotinia* species have been described since the last taxonomic revision, including *Sclerotinia antarctica*, *S. asari*, *S. atrostipitata*, *S. cirsii-spinosissimi*, *S. ginseng*, *S. glacialis*, *S. himalayensis*, *S. nivalis*, *S. pseudoplatani*, *S. subarctica*, *S. tetraspora*, *S. trillii*, and *S. verrucispora*. These species are evaluated here. Finally, several recommendations are made regarding how future taxonomic research on *Sclerotinia* should incorporate molecular data. We highlight potential obstacles and opportunities for this research, including the limitations of the internal transcribed spacer rDNA region (ITS) as a DNA barcode and the untapped potential of genomic data for the genus. By outlining the gaps that need to be addressed, this review charts a course toward a clearer understanding of taxonomic relationships among *Sclerotinia* species. This understanding will facilitate research into other aspects, such as pathogenicity and host generalism, and may ultimately contribute to improved management of the devastating diseases caused by these pathogens.

## Introduction

The genus *Sclerotinia* [MycoBank (MB)#4942] contains several plant pathogens that cause significant economic losses in agriculture (Bolton et al. 2006). The best-known species, *Sclerotinia sclerotiorum* [MB#212553], is estimated to cause annual losses exceeding 200 million dollars in the United States (Bolton et al. 2006). These numbers can be significantly affected by severe outbreaks, as seen in 2009, when *Sclerotinia* on soybean incurred an estimated cost of $560,149,000 (Peltier et al. 2012). Considering that these figures are neither global nor recent, the real impact of diseases caused by this fungus is certainly much greater. Given that *S. sclerotiorum* infects over 425 hosts (Derbyshire et al. 2022) and has a near-worldwide distribution, estimating the true economic cost is complicated, if not near impossible. *Sclerotinia* also contains at least two other plant pathogens, *S. minor* [MB#271273] and *S. trifoliorum* [MB#207563], which contribute to agricultural losses (O’Sullivan et al. 2021). Other *Sclerotinia* species are known, but their importance as plant pathogens remains uncertain (Svrček 1979, 1988; Sharma and Thind 1983; Wang and Wu 1983; Gamundi and Spinedi 1987; Baral 1989; Holst-Jensen and Schumacher 1994; Graf and Schumacher 1995; Wang et al. 1995; Saito 1997; Narumi et al. 2001; Winton et al. 2007; Senn-Irlet and Peter 2016).

Apart from its agricultural impacts, *Sclerotinia* and species from related genera in the family *Sclerotiniaceae* [MB#81363] present an interesting case study for host generalism. *Sclerotinia sclerotiorum*, *S. minor*, and *S. trifoliorum* all infect multiple plant species, similar to another generalist pathogen from *Sclerotiniaceae*, *Botrytis cinerea* [MB#217312] (Williamson et al. 2007). This presents an opportunity for molecular and genomic investigations to uncover insights into pathogenicity and host generalism. Some key pathogenicity genes within *Sclerotiniaceae* have already been explored using comparative genetics (Andrew et al. 2012), but this only scratches the surface of what could be learned from these pathogens. The knowledge gained from these kinds of studies extends beyond *Sclerotinia*, enhancing our overall understanding of plant pathogens and the mechanisms driving disease.

Despite its significant agricultural and scientific importance, the taxonomy of *Sclerotinia*, and particularly *S. sclerotiorum*, remains unresolved. Its taxonomic history is complex, and any contemporary evaluation must acknowledge and account for this. *Sclerotinia sclerotiorum* is recognised as the type species of *Sclerotinia*, which in turn typifies the family *Sclerotiniaceae*. This classification was first formalised by Whetzel (1945), when he introduced the family and rearranged several related genera. Understanding the complex history of *Sclerotinia* taxonomy requires a discussion of two pivotal publications: the rearrangements suggested by Whetzel (1945) and the monographic treatment by Kohn (1979). Whetzel’s work represents a convergence point for the histories of *S. sclerotiorum*, the genus *Sclerotinia*, and the family *Sclerotiniaceae*, while Kohn (1979) provides a clearer definition of the genus and its species. The current review traces the history of *Sclerotinia* taxonomy through these cornerstone publications and identifies the major gaps that persist. We examine the shifting circumscription of *Sclerotiniaceae*, from its original description to its current placement within *Leotiomycetes*, and we evaluate key publications and recent phylogenetic frameworks to clarify the core genera of the family. We also outline various morphological characters used to define species and review the status of *Sclerotinia* species described since Kohn (1979). Finally, we make recommendations for future work to establish a more robust framework for species delineation, which will help anchor research on these destructive plant pathogens.

## Taxonomic history of the genus *Sclerotinia*

**Figure 1.**
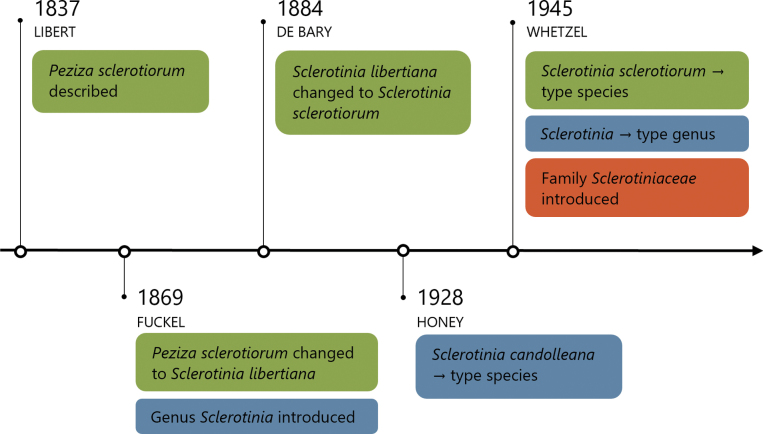
Timeline of *Sclerotinia* and *Sclerotiniaceae* taxonomy. Timeline depicting the taxonomic history of *Sclerotinia sclerotiorum* (green), the genus *Sclerotinia* (blue), and the family *Sclerotiniaceae* (orange), up until the rearrangement by Whetzel (1945). The symbol “→” denotes “declared,” and the taxonomists responsible for each decision are indicated beneath the corresponding dates.

The oldest name for *Sclerotinia sclerotiorum* is *Peziza sclerotiorum* [MB#168084], described in 1837 by Libert, who noted that its apothecia resembled ascomycetous cup fungi classified in *Peziza* (Fig. 1) (Willetts and Wong 1980). Thirty-three years later, Fuckel (1870) renamed *P. sclerotiorum* in honor of Libert as *Sclerotinia libertiana* when he introduced the genus *Sclerotinia* (Fig. 1), along with *S. candolleana* [MB#183164], *S. fuckeliana* [MB#211447], *S. tuberosa* [MB#163049], and *S. baccata* [MB#181805] (Fuckel 1870; Willetts and Wong 1980). Designation of nomenclatural types in publications only became mandatory from 1 January 1958 (Turland et al. 2018), resulting in species described before this time often lacking a type strain, and this is the case for *Sclerotinia*. Similarly, generic names were also not commonly typified, but *S. candolleana* was considered the generic type because it was listed first in Fuckel’s protologue (Honey 1928). De Bary (1887) considered *S. libertiana* illegitimate (nomen superfluum) and provided the new name *Sclerotinia sclerotiorum* as a result (de Bary 1887; Bolton et al. 2006).

Whetzel (1945) proposed major changes to *Sclerotinia* and related taxa when introducing the family *Sclerotiniaceae*. *Sclerotinia* was designated as the type of the family (Fig. 1), which included 15 genera (discussed in detail later). He proposed 12 species in *Sclerotinia* and designated *S. sclerotiorum* as the generic type. *Sclerotinia candolleana* was accommodated in the new genus *Ciborinia* as *C. candolleana* [MB#183164]. This decision was later challenged with the argument that *S. candolleana*, which Honey (1928) considered the type of *Sclerotinia*, should have remained in the genus *Sclerotinia* and *S. sclerotiorum* reclassified in a new genus. This move was subsequently made when Korf and Dumont (1972) introduced *Whetzelinia* and renamed *S. sclerotiorum* as *Whetzelinia sclerotiorum* [MB#325563]. However, Buchwald and Neergaard (1976) proposed conserving *Sclerotinia* with *S. sclerotiorum* (basionym: *Peziza sclerotiorum*) as the type species, which was approved by the Special Committee on Fungi and Lichens (Petersen 1978). This resulted in Whetzel’s classification that is still in use today, with *S. sclerotiorum* as the type species of *Sclerotinia*, which in turn is the type genus of *Sclerotiniaceae* (Whetzel 1945).

## Taxonomic history of the family *Sclerotiniaceae*

The higher-order taxonomy of *Sclerotiniaceae* is marked by historical instability. Whetzel (1945) classified *Sclerotiniaceae* in the class *Discomycetes* (order *Helotiales*), which contained all apothecia-producing fungi. *Sclerotiniaceae* more specifically accommodated the inoperculate *Discomycetes* (Whetzel 1945; Ainsworth et al. 1971). However, over time, it became clear that *Discomycetes* belonged to several unrelated lineages, leading to these fungi being reassigned to various taxonomic groups (Ainsworth et al. 2001). As a consequence, the order *Helotiales*, which includes the family *Sclerotiniaceae*, was moved to the class *Leotiomycetes* (Korf and Lizon 2000). Since then, the higher-level taxonomy of *Sclerotiniaceae* has remained consistent, with its position in *Ascomycota*, *Pezizomycotina*, *Leotiomycetes*, and *Helotiales* unchanged (Korf and Lizon 2000). Some publications still incorrectly list *Sclerotinia* species as *Discomycetes* (Saharan and Mehta 2008; Willbur et al. 2019; Otun et al. 2022), likely because the most cited review of *S. sclerotiorum* lists it in this class (Bolton et al. 2006).

Another significant change was when Holst-Jensen et al. (1997) split *Sclerotiniaceae* into two distinct families. Historically, all stromatic, stipitate discomycetes were grouped into *Sclerotiniaceae* (Whetzel 1945). However, several studies showed that the sclerotial and substratal stromatal taxa in *Sclerotiniaceae* likely represented separate lineages (Novak and Kohn 1991; Carbone and Kohn 1993). A phylogenetic evaluation of the family subsequently supported the two-lineage hypothesis (Holst-Jensen et al. 1997), triggering the formation of *Rutstroemiaceae* and the subsequent division of *Sclerotiniaceae* genera between these families (Holst-Jensen et al. 1997).

The higher-order reclassification of *Sclerotiniaceae* led to significant changes in the composition of its genera. When the family was first described, it included 15 genera: *Botryotinia* [MB#638], *Ciboria* [MB#1040], *Ciborinia* [MB#1042], *Coprotinia* [MB#1232], *Lambertella* [MB#2633], *Martinia* [MB#3007], *Monilinia* [MB#3249], *Ovulinia* [MB#3660], *Rutstroemia* [MB#4806], *Seaverinia* [MB#4990], *Septotinia* [MB#5006], *Sclerotinia* [MB#4942], *Streptotinia* [MB#5280], *Stromatinia* [MB#5288], and *Verpatinia* [MB#5724] (Whetzel 1945). Two additional genera, *Dumontinia* [MB#1715] and *Elliottinia* [MB#1760], were subsequently described in the family by Kohn (1979). Several changes were made following the introduction of the family *Rutstroemiaceae* (Holst-Jensen et al. 1997). *Sclerotiniaceae* retained the genera *Botryotinia*, *Ciboria*, *Ciborinia*, *Coprotinia*, *Dumontinia*, *Monilinia*, *Ovulinia*, *Sclerotinia*, and *Stromatinia*, and also gained five new genera: *Encoelia* [MB#1783], *Grovesinia* [MB#25807], *Myriosclerotinia* [MB#3376], *Pycnopeziza* [MB#4571], and *Valdensinia* [MB#5699] (Holst-Jensen et al. 1997). The genera *Lanzia* [MB#2639], *Poculum* [MB#4271], *Lambertella*, *Rutstroemia*, and *Verpatinia* were reclassified into *Rutstroemiaceae* (Holst-Jensen et al. 1997), with the latter three genera originally part of *Sclerotiniaceae* as described by Whetzel (1945).

The influential publications of Whetzel (1945), Kohn (1979), and Holst-Jensen et al. (1997) were the last to provide an in-depth evaluation of *Sclerotiniaceae*. Since 1997, several genera have been assigned to or removed from *Sclerotiniaceae* (Holst-Jensen et al. 2004; Narumi-Saito et al. 2006; Pärtel et al. 2017; Salgado-Salazar et al. 2018), although these changes have not been systematically evaluated—an endeavor that is outside the scope of this review. Instead, we rely on two recent phylogenetic studies of *Leotiomycetes* to assess the best current composition of *Sclerotiniaceae* (Ekanayaka et al. 2019; Johnston and Park 2025).

## *Sclerotiniaceae* in the era of phylogenetics

The three foundational publications of Whetzel (1945), Kohn (1979), and Holst-Jensen et al. (1997) collectively provide a “core list” of genera that have been central to past conceptions of the family. The 19 genera are *Botrytis* (previously *Botryotinia* (Hyde et al. 2014)), *Ciboria*, *Ciborinia*, *Coprotinia*, *Dumontinia*, *Elliottinia*, *Encoelia*, *Grovesinia*, *Martininia* (previously *Martinia* (Dumont and Korf 1970)), *Monilinia*, *Myriosclerotinia*, *Ovulinia*, *Pycnopeziza*, *Sclerotinia*, *Seaverinia*, *Septotinia*, *Streptotinia*, *Stromatinia*, and *Valdensia* (previously *Valdensinia* (Johnston et al. 2014)). This historical core serves as the anchor against which we evaluate the recent phylogenetic publications by Ekanayaka et al. (2019), Johnston et al. (2019), and Johnston and Park (2025).

**Figure 2.**
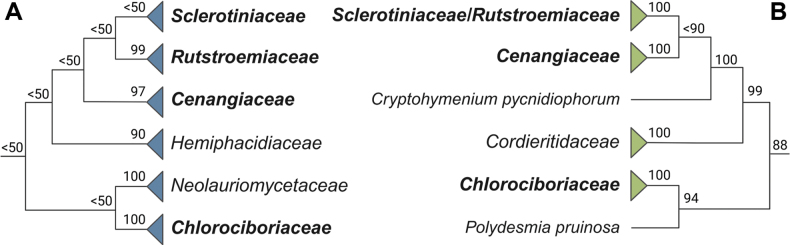
Comparison of sclerotinioid clades from recent phylogenetic studies. Side-by-side comparison of the “*Sclerotiniales*” and “sclerotinioid” clades identified by Ekanayaka et al. 2019 (**A**, in blue) and Johnston et al. 2019 (**B**, in green). Taxa present in both analyses are indicated in bold, and bootstrap values are those reported in the respective publications.

The multilocus and phylogenomic analyses of Ekanayaka et al. (2019) and Johnston et al. (2019)—updated by Johnston and Park (2025)—offer the most up-to-date insights into the classification of *Sclerotiniaceae* within *Leotiomycetes*. Ekanayaka et al. (2019) produced a maximum-likelihood tree from 482 strains representing 187 *Leotiomycetes* species based on sequence data from five regions, including the internal transcribed spacer rDNA region (ITS), the 28S nuclear ribosomal large subunit (LSU), the 18S nuclear ribosomal small subunit (SSU), and the partial RNA polymerase II core subunit (*rpb*2) and translation elongation factor 1-alpha (*tef*1-α) gene regions. They observed a “Sclerotiniales” clade that included the families *Cenangiaceae*, *Chlorociboriaceae*, *Hemiphacidiaceae*, *Neolauriomycetaceae*, *Rutstroemiaceae*, and *Sclerotiniaceae* (Fig. 2). Johnston et al. (2019) used three approaches to assess the phylogenetic classification of *Leotiomycetes*: a phylogenomic tree based on 3,156 single-copy genes from 51 *Leotiomycetes* species, a 15-gene phylogeny of 259 selected species, and an ITS phylogeny that included taxa not represented in the other analyses, with a focus on ex-type specimens of generic type species. They identified a “sclerotinioid” clade comprising four strongly supported subclades, namely a Sclerotiniaceae–Rutstroemiaceae clade, a Cenangiaceae clade, a Cordieritidaceae clade, and a Chlorociboriaceae clade (Fig. 2). This structure is broadly similar to the “Sclerotiniales” clade identified by Ekanayaka et al. (2019), which also includes *Cenangiaceae*, *Chlorociboriaceae*, *Rutstroemiaceae*, and *Sclerotiniaceae*. However, the additional families included within this grouping differ between the studies (Fig. 2). Recently, the ITS and multigene phylogenies of Johnston et al. (2019) were updated to incorporate newly available data and recent taxonomic changes (Johnston and Park 2025). Although some differences exist between the sclerotinioid clades recovered in the two studies, the broad structure is unchanged. Notably, the clade containing *Piceomphale* and ‘*Cenangium* acuum,’ which was treated as *Rutstroemiaceae* by Johnston et al. (2019), is now considered sister to the monophyletic *Sclerotiniaceae* and *Rutstroemiaceae* (Johnston and Park 2025).

A comparison between the historical core list and the genera included in recent phylogenetic reconstructions reveals notable differences in family composition. The two studies identified 36 genera as belonging to *Sclerotiniaceae* (Table 1) (Ekanayaka et al. 2019; Johnston and Park 2025). Twenty-six genera were placed in *Sclerotiniaceae* by both studies, and an additional four genera were identified as *Sclerotiniaceae* by one of the two publications but were not evaluated in the other (Table 1). Six genera showed conflicting placements, with *Banksiamyces*, *Clarireedia*, *Coprotinia*, *Martininia*, *Poculum*, and *Scleromitrula* assigned to *Sclerotiniaceae* by Ekanayaka et al. (2019) but placed in *Helotiales* incertae sedis or *Rutstroemiaceae* by Johnston and Park (2025) (Table 1, Suppl. material 1: table SS2). Of the 19 historical genera, 18 are included in *Sclerotiniaceae* by one or both studies, with the notable exception of *Encoelia*, which was placed in *Hemiphacidiaceae* by Ekanayaka et al. (2019) and in *Cenangiaceae* by Johnston and Park (2025).

**Table 1.** Genera of *Sclerotiniaceae* in recent treatments. Summary of 36 *Sclerotiniaceae* genera as treated by Ekanayaka et al. 2019 and Johnston and Park 2025. Asterisks indicate genera that are part of the historical list collated from Whetzel (1945), Kohn (1979), and Holst-Jensen et al. (1997).

Assigned to *Sclerotiniaceae* by both publications	Only evaluated in Ekanayaka et al. (2019)	Only evaluated in Johnston and Park (2025)	Conflicting assignments
* Amphobotrys *	Septotinia*	* Microstrobilinia *	* Banksiamyces *
Botrytis*		* Schroeteria *	* Clarireedia *
Ciboria*		* Septotis *	Coprotinia*
Ciborinia*			Martininia*
* Cristulariella *			* Poculum *
* Cudoniopsis *			* Scleromitrula *
Dumontinia*			
Elliottinia*			
Grovesinia*			
* Haradamyces *			
* Kohninia *			
Monilinia*			
* Mycopappus *			
* Myrioconium *			
Myriosclerotinia*			
Ovulinia*			
* Phaeosclerotinia *			
* Pseudociboria *			
Pycnopeziza*			
* Redheadia *			
* Sclerencoelia *			
Sclerotinia*			
Seaverinia*			
Streptotinia*			
Stromatinia*			
Valdensia*			

The recent phylogenetic studies of Ekanayaka et al. (2019) and Johnston and Park (2025) add an additional 18 genera to the original core genera defined by Whetzel (1945), Kohn (1979), and Holst-Jensen et al. (1997). This clearly shows that the 19 core genera provide only a framework and reiterates the need for an extensive review of all possible *Sclerotiniaceae* genera. In the interim, MycoBank provides a record of genera associated with *Sclerotiniaceae*, listing a total of 47 genera (Suppl. material 1: table SS1), 12 of which are not represented in current phylogenetic studies (Suppl. material 1: table SS2). Future revisions should focus on obtaining reference material for these unsequenced genera to confirm their classification in *Sclerotiniaceae* (Suppl. material 1: table SS2).

Despite their value, phylogenetic approaches have not resolved all relationships within the sclerotinioid clade, leaving the placement of several genera uncertain. One example is the shifting placement of *Verpatinia*. In 1997, the genus was synonymised with *Scleromitrula* [MB#4935], assigning it to *Sclerotiniaceae* (Schumacher and Holst-Jensen 1997). Yet later that same year, Holst-Jensen et al. (1997) transferred *Verpatinia* to *Rutstroemiaceae*, contradicting the earlier treatment. More recently, Ekanayaka et al. (2019) showed that the generic type, *Scleromitrula shiraiana* (isolate Hirayama062001), resolved within *Sclerotiniaceae*. In contrast, Johnston et al. (2019), analyzing the same isolate along with *Scleromitrula spiraeicola* and *Verpatinia calthicola*, placed *Scleromitrula* in *Rutstroemiaceae* and *Verpatinia* in *Sclerotiniaceae*—the reverse of the findings of Ekanayaka et al. (2019). The revised phylogenies presented in Johnston and Park (2025) lack data on *Verpatinia* and thus cannot resolve this discrepancy. These conflicting placements underscore the instability of phylogenetic reconstructions for this group and highlight the need for further study and taxonomic revision. Both Johnston et al. (2019) and Johnston and Park (2025) demonstrated that although *Sclerotiniaceae* is monophyletic, *Rutstroemiaceae* is polyphyletic. Achieving monophyly would therefore require splitting *Rutstroemiaceae* into several smaller families, an approach that resolves phylogenetic conflict but increases taxonomic complexity (Johnston et al. 2019). In contrast, Johnston and Park (2025) discuss whether *Sclerotiniaceae* should be broadened to include *Rutstroemiaceae*, an approach that would circumvent the need to split *Rutstroemiaceae* into multiple families.

Although phylogenetic approaches provide valuable taxonomic insight, the issues discussed above highlight their limitations. These limitations are likely due, at least in part, to incomplete taxon sampling. Moving forward, a more complete and taxonomically inclusive understanding of *Sclerotiniaceae* will require systematic sampling of undersampled and candidate genera or species where type specimens or species are not available. To resolve this issue, a concerted effort must be made to recollect or obtain authentic material from collections and generate sequences from them. Guidelines for prioritising this sampling and sequence generation were discussed by Johnston et al. (2019).

## The current taxonomy of *Sclerotinia*

### *Sclerotinia* species accepted by Kohn (1979)

Species delineation in *Sclerotinia* has been the subject of much debate. Before 1979, 279 *Sclerotinia* species were described. Their taxonomy was revised by Kohn (1979), with only three species—*S. sclerotiorum*, *S. minor*, and *S. trifoliorum*—being accepted. Wherever possible, Kohn (1979) examined specimens and their published protologues, which led to the synonymization of 21 names with the three accepted species. Two hundred and ten names were removed from *Sclerotinia* and transferred to other genera (Kohn 1979). While evaluating these 279 names, she also introduced two genera, *Dumontinia* and *Elliottinia*, to accommodate *Sclerotinia tuberosa* and *Sclerotinia kerneri*, respectively (Kohn, 1979). Of the 279 names revised, 25 species could not be adequately evaluated with the available information, and their identities remain uncertain.

No living type material was available for the three species accepted by Kohn (1979), leading to the lectotypification of *S. sclerotiorum* and the neotypification of *S. minor* and *S. trifoliorum*. Below, we include a nomenclator for Kohn’s accepted species:

#### Sclerotinia sclerotiorum

Taxon classificationFungiHelotialesDiscomycetes

(Lib.) de Bary, Vergleichende Morphologie und Biologie der Pilze Mycetozoen und Bacterien: 56 (1884) [MB#212553]. Typus: Libert, ad Sclerotium tectum, Aestate, Crypt. Ard. 326 [BR: LIBERT, CRYPT. ARD. 326, lectotype of S. sclerotiorum].

4BF9C4DF-9A03-5118-A71D-29A844F2FE44

≡ Peziza sclerotiorum Lib., Plantae Cryptogamae, quas in Arduenna collegit Fasc. 4: no. 326 (1837) [MB#168084].
≡ Helotium sclerotiorum (Lib.) Fuckel, Fungi Rhenani Exsiccati, Supplementi Fasc. 4: no. 1861 (1866) [MB#565328].
= Peziza coemansii J.J. Kickx, Flore Cryptogamique des Flandres 1: 485 (1867) [MB#207760].
= Peziza kauffmanniana Tikhom. (1868) [MB#118315].
≡ Sclerotinia libertiana Fuckel, Jahrbücher des Nassauischen Vereins für Naturkunde 23–24: 331 (1870) [MB#191471].
≡ Phialea sclerotiorum (Lib.) Gillet, Champignons de France. Les Discomycètes 4: 98 (1881) [MB#177868].
= Sclerotinia postuma Berk. & Wilson. Gardeners’ Chronicle 20:333 (1883) [MB#N/A].
≡ Hymenoscyphus sclerotiorum (Lib.) W. Phillips, A manual of the British Discomycetes: 115 (1887) [MB#461963].
= Sclerotinia ficariae Rehm, Rabenhorst’s Kryptogamen-Flora, Pilze - Ascomyceten 1(3): 815 (1893) [MB#215234].
= Sclerotinia henningsiana Kirschst., Verh. Bot. Ver. Prov. Brandenb.: XXVII (1898) [MB#213131].
= Sclerotinia opuntiarum Speg., Anales de la Sociedad Científica Argentina 50: 37 (1900) [MB#188934].
≡ Sclerotinia sclerotiorum (Lib.) de Bary var. *opuntiarum* (Speg.) Alippi. [MB#350337].
= Sclerotinia moelleriana Henn., Hedwigia 41: 27 (1902) [MB#194169].
= Sclerotinia wisconsinensis Rehm, Annales Mycologici 6 (4): 317 (1908) [MB#174190].
= Sclerotinia matthiolae Lendn., Bull. Soc. bot. Genève: 7–9, 21 (1917) [MB#270652].
= Sclerotinia sclerotiorum f. *orobanches* Naras. & Thirum., Phytopath. Z.: 426 (1954) [MB#347601].
≡ Whetzelinia sclerotiorum (Lib.) Korf & Dumont, Mycologia 64: 250 (1972) [MB#325563].
= Sclerotinia riograndensis Rick (1931) [MB#254403].
= Sclerotinia galeopsidis Velen., Monographia Discomycetum Bohemiae: 227 (1934) [MB#265752].
= Sclerotinia caudata Velen., Novitates mycologicae novissimae: 129 (1947) [MB#290797].
= Sclerotinia xanthorrhoeae G.W. Beaton & Weste, Transactions of the British Mycological Society 68 (1): 73 (1977) [MB#323268]
= Rutstroemia homocarpa P. Karst. Bidrag Till Kannedom Om Finlands Natur Och Folk 19: 107 (1871) [MB#161056]

#### Sclerotinia minor

Taxon classificationFungiHelotialesSclerotiniaceae

Jagger, J. Agric. Res. 20: 333 (1920) [MB#271273]. Typus: Porter, 1.IX.1974, on Arachis hypogaea, Southampton Co., Virginia, CUP 58237.

BB27C3E3-CEC6-59CF-B9B0-0AA7B2E4C182

*= Sclerotinia intermedia* Ramsey, Phytopathology 14:324 (1924) [MB#268041].
*= Sclerotinia sativa* Drayton & Groves, Mycologia 35: 526 (1943) [MB#290804].

#### Sclerotinia trifoliorum

Taxon classificationFungiHelotialesSclerotiniaceae

Erikss., Kungliga Landtbraksakoemiens handlingar och tidskrift: 28 (1880) [MB#207563]. Typus: Jakob Eriksson, IX.1878 [s-Svensk Svamp Herb. 1a, neotype of Sclerotinia trifoliorum].

C1C713E8-7B68-5A04-AF2D-79C332129B15

*= Peziza ciborioides* Hoffm. Fungi Europaei Exsiccati: 619 (1864) [MB#N/A].
*= Sclerotinia bryophila* Kirschst. Annales Mycologici 36: 381 (1938) [MB#259056].

### Morphological characterization of *Sclerotinia*

**Figure 3.**
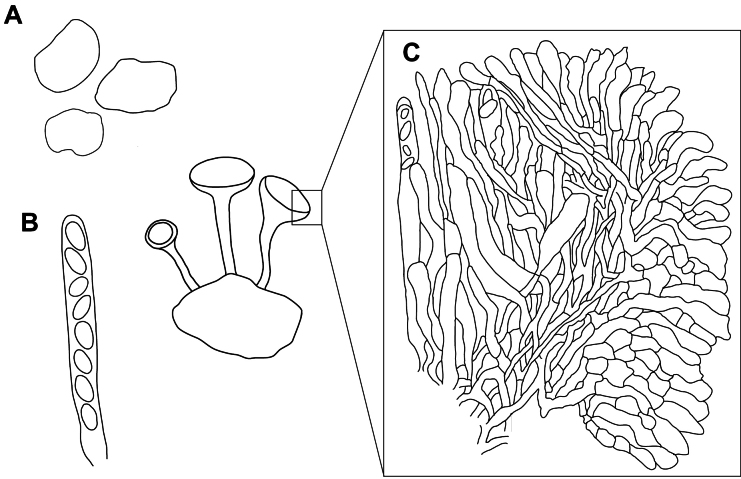
Morphological features delimiting *Sclerotinia*. General morphological characteristics delimiting *Sclerotinia* as defined by Whetzel (1945) and Kohn (1979). The genus is defined by the presence of definite, tuberoid sclerotia not incorporating host tissue (**A**), single-celled, hyaline ascospores (**B**), and cells of the outer excipulum of the apothecium that are globose and oriented perpendicularly to the apothecial surface (**C**). Illustration for (**C**) is replicated from Kohn (1979), fig. 4.

Before Kohn (1979), standard criteria for characterising *Sclerotinia* and differentiating between species had not been established. Species were typically distinguished based on the size and shape of ascospores or sclerotia (Korf and Dumont 1972), but other characters were used inconsistently. One of the most valuable elements of Kohn’s monograph was her extensive review of the morphological characters informative in delimiting *Sclerotinia* species and in defining the genus based on these. Whetzel’s (1945) initial description of the genus was limited to species that produce definite, tuberoid sclerotia, have single-celled, hyaline ascospores, and lack a functional conidial state. Kohn (1979) further restricted the genus by including only those species in which the cells of the outer excipulum of the apothecium are globose and oriented perpendicularly to the apothecial surface (Fig. 3).

Kohn (1979) distinguished between *S. sclerotiorum*, *S. minor*, and *S. trifoliorum* based on characteristics of their ascospores, apothecia, and sclerotia in agar culture (Table 2). *Sclerotinia trifoliorum* is the only species that exhibits ascospore dimorphism, with a segregation of large and small ascospores that is typically observed in a 4:4 ratio. *Sclerotinia trifoliorum* also has tomentum hyphae that extend beyond the sclerotial rind, a character that is absent in the other species. Ascospores of *S. sclerotiorum* contain two nuclei, whereas those of *S. minor* and *S. trifoliorum* contain four. *Sclerotinia sclerotiorum* and *S. trifoliorum* typically produce small numbers of large sclerotia (>2 mm) on the growing tips of hyphae at the periphery of the colony, whereas *S. minor* forms many smaller sclerotia (0.5–2 mm) that develop laterally on hyphae dispersed throughout the colony. *Sclerotinia sclerotiorum* and *S. minor* can also be differentiated based on the cell shape of the ectal excipulum of the stipe (Kohn, 1979), with cells being shorter (textura porrecta) in *S. sclerotiorum* but elongated (textura prismatica) in the other two species. Shortly after Kohn’s monograph, Willetts and Wong (1980) also accepted *S. sclerotiorum*, *S. minor*, and *S. trifoliorum* but used additional characters and traits such as colony growth rate, host range, and mycelial interaction to delineate these species. In recent years, the recognised host range of *S. sclerotiorum* has expanded significantly (Derbyshire et al. 2022), reducing the usefulness of host specificity as a diagnostic characteristic for species delineation. Nevertheless, a selection of the traits used by Kohn (1979) and Willetts and Wong (1980) is presented in Table 2.

**Table 2.** A selection of diagnostic characters for the three core *Sclerotinia* species. These are some of the characters used by Kohn (1979) and Willetts and Wong (1980) to recognise and distinguish *S. sclerotiorum*, *S. minor*, and *S. trifoliorum*. Although representative, this list is not exhaustive.

Character		*S. sclerotiorum*	*S. minor*	*S. trifoliorum*
Number of nuclei per ascospore		2	4	4
Haploid chromosome numbers (n)		16*	4	8
Ascospore dimorphism		No	No	Yes
Sclerotia in culture		Fewer, large	Many, small	Fewer, large
Sclerotial formation in culture		On growing tips of hyphae, at colony periphery	Laterally on hyphae, throughout colony	On growing tips of hyphae, at colony periphery
Sclerotial rind		No tomentum hyphae	No tomentum hyphae	Tomentum hyphae extend beyond rind
Sclerotial patterning in culture		One, two, or more concentric rings may be discernible. Single ring often near edge of petri dish	Irregularly arranged throughout cultures	As for *S. sclerotiorum*, but sometimes irregularly distributed
Electrophoretic patterns		Distinctive for this fungus	Distinctive	Distinctive, intermediate in some respects between *S. sclerotiorum* and *S. minor*
Ectal excipulum (apothecia)	Cell shape	Textura prismatica, sometimes bound in gel	Textura prismatica, sometimes bound in gel, but more often only cells at margin bound in gel	Textura prismatica
	Orientation	Perpendicular to apothecial surface	Perpendicular to apothecial surface	Perpendicular to apothecial surface
	Outermost cells	Margin consists of textura porrecta, outermost excipular cells sometimes give rise to 1–2 celled tomentum hyphae	Outermost excipular cells often give rise to 1–2 celled tomentum hyphae	Margin consists of textura porrecta, outermost excipular cells often give rise to 1–2 celled tomentum hyphae
Ectal excipulum (stipe)	Cell shape	Textura porrecta	Textura prismatica	Textura prismatica
	Orientation		Turning out perpendicular to stipe axis	Oriented parallel to stipe axis
	Outermost cells	Give rise to one-celled tomentum hyphae, which turn out perpendicularly to stipe axis	Give rise to 1–2 celled tomentum hyphae, which sometimes group in fascicles	Give rise to one-celled tomentum hyphae, which turn out perpendicularly to stipe axis
Mycelial	Growth rate on solid agar media at 26 °C	Fast	Not documented	Slow – about half that of *S. sclerotiorum*
	Formation of haptera at edges of petri dishes	Produced infrequently	Variable but usually produced infrequently	Produced frequently
Mycelial	Aerial mycelia	Moderate to abundant amounts of aerial mycelium giving white appearance to plates	Moderate aerial mycelium	Sparse aerial mycelium

*Although Kohn (1979) and Willetts and Wong (1980) list the haploid chromosome number as 8, genomic data has shown that this species possesses 16 chromosomes. This inconsistency is explained by a newly discovered phenomenon whereby *S. sclerotiorum* divides its haploid chromosomes between two nuclei (Tian et al. 2025).

### *Sclerotinia* species described since Kohn (1979)

Kohn (1979) brought much-needed stability to *Sclerotinia* taxonomy through her restriction of the genus to three core species. Thirteen species have subsequently been described, namely *S. antarctica* [MB#130648], *S. asari* [MB#108507], *S. atrostipitata* [MB#133749], *S. cirsii-spinosissimi* [MB#516616], *S. ginseng* [MB#414139], *S. glacialis* [MB#363084], *S. himalayensis* [MB#107281], *S. nivalis* [MB#442361], *S. pseudoplatani* [MB#323267], *S. subarctica* [MB#586496], *S. tetraspora* [MB#362729], *S. trillii* [MB#47465], and *S. verrucispora* [MB#135633] (Svrček 1979, 1988; Sharma and Thind 1983; Wang and Wu 1983; Gamundi and Spinedi 1987; Baral 1989; Holst-Jensen and Schumacher 1994; Graf and Schumacher 1995; Wang et al. 1995; Saito 1997; Narumi et al. 2001; Winton et al. 2007; Senn-Irlet and Peter 2016). Most of these new species were described based solely on morphology, and many lack DNA sequence data or living strains deposited in culture collections. Here, we review and comment on the species introduced since Kohn (1979).

#### Sclerotinia antarctica

Taxon classificationFungiHelotialesSclerotiniaceae

Gamundí & Spinedi, Mycotaxon 29: 84 (1987) [MB#130648].

C117AE34-C721-5A5A-BCC7-EB945A577E46

##### Type.

ANTARCTICA • Antarctic Peninsula: Danco Base Primavera (Cierva Point), on leaves, glumes, or scapes of *Deschampsia antarctica*, 6 Feb 1986, H.A.Spinedi (holotype: LPS 44123).

##### Discussion.

*Sclerotinia antarctica* was described from culms of Antarctic hair grass (*Deschampsia antarctica*) in Antarctica (Gamundi and Spinedi 1987). The species produces definite sclerotia, has single-celled, hyaline ascospores, and lacks a conidial state. The cells of the outer excipulum of the apothecium are globose and appear to be oriented perpendicularly to the apothecial surface (Gamundi and Spinedi 1987), which is a defining character of *Sclerotinia* species (Kohn 1979). The authors considered *S. antarctica* most similar to *Sclerotinia borealis* and suggested that they might represent a distinct genus. Kohn (1979) placed *S. borealis* in *Myriosclerotinia*, but this treatment was subsequently rejected (Schumacher and Kohn 1985). DNA sequence data are required to confirm the taxonomic placement of *S. antarctica*.

#### Sclerotinia asari

Taxon classificationFungiHelotialesSclerotiniaceae

Y. Wu & C.R. Wang, Acta Phytotax Sin: 10 (1983) [MB#108507].

C43324F5-23E8-55D8-88FF-4CACF75624B3

##### Type.

CHINA • Liaoning: Shenyang, on *Asarum heterotropoides* var. *mandshuricum*, Y.Wu & C.R.Wang (holotype: S_1_(1), Shenyang Agricultural College Mycological Herbarium).

##### Discussion.

*Sclerotinia asari* has a dual history. Kohn (1979) noted that the epithet “asari” was used by Whetzel for isolates he collected “near *Asarum*.” However, this name was never published, and these isolates were later considered to belong to *Dumontinia tuberosa*Kohn (1979). *Sclerotinia asari* was later formally described on *Asarum heterotropoides* (Wang & Wu, 1983). No DNA sequence data are available for this species, but Kohn et al. (1988) conducted an RFLP comparison and concluded that *S. asari* is distinct from the three core *Sclerotinia* species (Kohn et al. 1988). However, it remains impossible to determine how this species fits within *Sclerotinia* or the broader family without DNA sequence data.

#### Sclerotinia atrostipitata

Taxon classificationFungiHelotialesSclerotiniaceae

Svrček, Czech Mycol 42 (3): 145 (1988) [MB#133749].

BC004ED8-27D9-5DB6-8AF6-4571C181573A

##### Type.

CZECH REPUBLIC • Central Bohemia: Prague, Žižkov, a single apothecium on *Ceratodon*, growing in pot with rooting *Evonymus*, 25 March 1986, M.Svrčková (holotype: PRM 948329).

##### Discussion.

*Sclerotinia atrostipitata* was described among the rhizoids of living stems of the moss *Ceratodon purpureus* (Svrček 1988). The species was described based on morphology and meets only some of the criteria for inclusion in *Sclerotinia*. It produces definite sclerotia and possesses hyaline ascospores. However, it is unclear whether the ascospores are single-celled. Species of *Sclerotinia* are defined by cells of the outer excipulum of the apothecium being globose and oriented perpendicularly to the apothecial surface. In *S. atrostipitata*, these cells are described as globose or subglobose, and there is no mention of their orientation. Additional work is required to evaluate the taxonomic placement of *S. atrostipitata*.

#### Sclerotinia cirsii-spinosissimi

Taxon classificationFungiHelotialesSclerotiniaceae

Senn-Irlet, Ascomycete.org 8 (5): 236 (2016) [MB#516616].

41DE649C-2F59-5870-93B2-FFEC1B649D4C

Botrytis cirsii-spinosissimi (Senn-Irlet) Baral, Index Fungorum 454: 2 (2020) [MB#556845]. Synonym.

##### Type.

SWITZERLAND • Uri: Attinghausen, 31 August 1996, B.Senn-Irlet (holotype: BSI 96/32 (ZT)).

##### Discussion.

*Sclerotinia cirsii-spinosissimi* was described using phylogenetic and morphological data. In a phylogeny based on the ITS locus, two putatively new taxa were identified and temporarily named *Sclerotinia* sp. 1 and *Sclerotinia* sp. 2 (Holst‐Jensen et al. 1998). *Sclerotinia* sp. 2 was later described as *S. cirsii-spinosissimi*, distinguishable from other *Sclerotinia* species based on ITS sequences and morphological features, including sclerotial size and the size, shape, and presence of lipid guttules in the ascospores (Senn-Irlet and Peter 2016). Based on ITS phylogenies, Holst‐Jensen et al. (1998) and Senn-Irlet and Peter (2016) showed that *S. cirsii-spinosissimi* groups most closely with *Sclerotinia borealis* (Senn-Irlet and Peter 2016), which Kohn (1979) considered to belong to *Myriosclerotinia* but was later regarded as doubtful by Schumacher and Kohn (1985). Baral and Quijada (2020) subsequently introduced the new combination *Botrytis cirsii-spinosissimi* for this species. This decision was based on morphological characters, namely the presence of host remnants in the sclerotial medulla and vacuolar bodies in the living paraphyses, as well as analysis of ITS sequences (H.O. Baral, personal communication, November 2025). The ITS sequence of the *S. cirsii-spinosissimi* type strain (GQ848548.1) shares 98.3–98.7% BLAST similarity with many *Botrytis cinerea* sequences, and phylogenetic analyses of ITS data suggest that it is more closely related to *Botrytis* than to *Sclerotinia* (H.O. Baral, personal communication, November 2025). The phylogenetic work conducted to date on *S. cirsii-spinosissimi* has relied solely on ITS sequence data, as this is the only region for which sequences are currently available. Additional gene regions are required to clarify this species’ relationships within *Sclerotiniaceae*.

#### Sclerotinia ginseng

Taxon classificationFungiHelotialesSclerotiniaceae

C.R. Wang, C.F. Chen & J. Chen, Acta Mycol Sin: 187 (1995) [MB#414139].

19E07A84-88DA-50D5-9968-E826A56E8030

##### Type.

CHINA • Liaoning: Shenyang, from roots of *Panax ginseng*, 4 Sept 1980, C.R.Wang, C.F.Chen & J.Chen, (holotype: Wang 800719, Shenyang Agricultural University).

##### Discussion.

*Sclerotinia ginseng* was introduced by Wang et al. (1995) and was distinguished from *S. sclerotiorum*, *S. minor*, *S. trifoliorum*, and *S. asari* based on morphology, soluble protein banding patterns, and the electrophoretic patterns of two pectinases, polygalacturonase and pectinesterase. The species has a narrower host range than other *Sclerotinia* species, primarily infecting ginseng, showing no pathogenicity on *Asarum* and only mild pathogenicity on soybean (Wang et al. 1995). While Wang et al. (1995) did not clarify whether the cells of the outer excipulum of the apothecium of *S. ginseng* are globose and oriented perpendicularly to the apothecial surface, all other defining features of *Sclerotinia* species are present, and the morphological and electrophoretic evidence supports recognition of *S. ginseng* as an independent species.

#### Sclerotinia glacialis

Taxon classificationFungiHelotialesSclerotiniaceae

F. Graf & T. Schumacher, Mycol Res 99 (1): 113 (1995) [MB#363084].

8E3147B5-0916-578E-AF18-3411217E6171

##### Type.

SWITZERLAND • Grisons: Radant, 28 July 1991, F.Graf (holotype: Z-ZT 16657).

##### Discussion.

*Sclerotinia glacialis* was isolated and described from glacier buttercups (*Ranunculus glacialis*) using morphological characters. It was distinguished from the three species accepted by Kohn’s (1979) based on ascospore size (8–10 µm × 22–27 µm in *S. glacialis* vs. 4–9 µm × 8–20 µm in the other species) and the presence of four-spored asci (vs. eight) (Graf and Schumacher 1995). Although an ITS phylogeny showed *S. glacialis* clustered with another newly described species, *S. nivalis* (Senn-Irlet and Peter 2016), the lack of bootstrap support casts doubt on this finding. However, the cells of the ectal excipulum conform to those of *Sclerotinia sensu*Kohn (1979), supporting *S. glacialis* as a true *Sclerotinia* species. Additional phylogenetic work is needed to support its placement in *Sclerotinia* and to clarify its relationship to other species in the genus.

#### Sclerotinia himalayensis

Taxon classificationFungiHelotialesSclerotiniaceae

M.P. Sharma & K.S. Thind, Bibl Mycol. 91: 182 (1983) [MB#107281].

BEC620A8-27FC-5E36-8E71-385FBD424826

##### Type.

INDIA • Himachal Pradesh: on buried seed of *Polygonium*, M.P.Sharma & K.S.Thind.

##### Discussion.

*Sclerotinia himalayensis* was described by Sharma and Thind (1983). However, repeated attempts to access the original publication were unsuccessful, and it is therefore not possible to evaluate the status of this species at present. Type information is taken from MycoBank (specimen record #68888).

#### Sclerotinia nivalis

Taxon classificationFungiHelotialesSclerotiniaceae

I. Saito, Mycoscience 38 (2): 229 (1997) [MB#442361].

2D6A6D23-3796-54F5-8AB8-FCBC340483E9

##### Type.

JAPAN • Makubetsu-cho: Hokkaido, on *Arctium lappa* (edible burdock), 15 May 1982, I.Saito (holotype: ISNAD 23-1, Herbarium of the Faculty of Agriculture, Hirosaki University #24055).

##### Discussion.

*Sclerotinia nivalis* was described as the causal agent of snow mold by Saito (1997). Isolates of this species were originally obtained in Japan and assigned to *Sclerotinia intermedia* (Tochinai 1958; Saito 1997)*. Sclerotinia intermedia* was subsequently synonymised with *S. sclerotiorum* (Purdy 1955). A re-evaluation of isolates collected between 1981 and 1985 confirmed that these represented a separate species, which was named *S. nivalis* (Saito 1997). This species was distinguished from the three core *Sclerotinia* species by its intermediate sclerotial size in culture, binucleate ascospores, molecular mass differences of certain sclerotial proteins, and patterns of esterase isozymes (Saito 1997). The RFLP study by Kohn et al. (1988) which supported the designation of *S. sclerotiorum*, *S. minor*, and *S. trifoliorum* as distinct species, also included an isolate of *S. nivalis* and showed that *S. nivalis* and *S. asari* are distinct from the three core *Sclerotinia* species. However, this study did not resolve the precise relationships among these taxa.

#### Sclerotinia pseudoplatani

Taxon classificationFungiHelotialesSclerotiniaceae

Svrček, Czech Mycol 33 (4): 205 (1979) [MB#323267].

BD575DEA-5985-5E4C-A957-1BC438D6FC4E

##### Type.

CZECH REPUBLIC • Southern Bohemia, Gabreta Mountains, Šumava, on the summit of Zátoňská hora mountain near Lenora, at around 1000 meters above sea level, 17 May 1975, collected by J.Kubická & M.Svrček (holotype: PRM 820982).

##### Discussion.

*Sclerotinia pseudoplatani* was described on decaying leaves of *Acer pseudoplatanus* and was considered a close relative of *S. candolleana* (Svrček 1979), which has since been transferred to *Ciborinia* as *Ciborinia candolleana* [MB#285221]. It is not clear from the original description whether the cells of the apothecial excipulum are globose and oriented perpendicularly to the apothecial surface, which is typical for *Sclerotinia*. On this basis, *S. pseudoplatani* is unlikely to belong in *Sclerotinia*.

#### Sclerotinia subarctica

Taxon classificationFungiHelotialesSclerotiniaceae

(nom. inval., Art. 36.1(a) (Shenzhen)) L.M. Winton, A.L. Krohn & R.H. Leiner ined.: 1077 (2007) [MB#586496].

03920E54-EDBE-5F30-9577-4DE373B78FFF

##### Type.

None, because no formal species description exists.

##### Discussion.

Holst‐Jensen et al. (1998) identified two putative new taxa in an ITS phylogeny, which were temporarily named *Sclerotinia* sp. 1 and *Sclerotinia* sp. 2. The latter was subsequently described as *S. cirsii-spinosissimi* by Senn-Irlet and Peter (2016). Later, a report of *Sclerotinia* sp. 1 infecting lettuce, cabbage, beans, and potatoes in Alaska was published by Winton et al. (2006). This taxon was subsequently referred to as *S. subarctica* in a publication describing microsatellite markers for the species, and the authors stated that a “formal species description of *S. subarctica* is in preparation” (Winton et al. 2007). However, it appears that this species description was never realised, although multiple publications have since reported infection by *S. subarctica* in new regions and on new hosts (Clarkson et al. 2010, 2017; Brodal et al. 2016; Leyronas et al. 2018). In light of the widespread and continued use of the provisional name *Sclerotinia subarctica*, a formal taxonomic revision and valid species description are needed to ensure nomenclatural stability.

#### Sclerotinia tetraspora

Taxon classificationFungiHelotialesSclerotiniaceae

Holst-Jensen & T. Schumach., Mycol Res 98: 926 (1994) [MB#362729].

FFB11112-74C7-5232-AA34-312400448F84

##### Type.

NORWAY • Hedmark: Engerdal, in a swamp near Galtsjøen, apothecia growing from sclerotium, inside the central cavity of dead *Rubus chamaemorus*, 21 June 1989, A.Holst-Jensen & T.Schumacher (holotype: University herbarium of Oslo 89/106).

##### Discussion.

*Sclerotinia tetraspora* was described from *Rubus chamaemorus*, a species of flowering plant in the rose family (Holst-Jensen and Schumacher 1994). The species was distinguished from other *Sclerotinia* species based on its four-spored asci and unique RFLP patterns. Cells of the ectal excipulum are described as “isodiametric to slightly elongate,” as opposed to globose, but are oriented perpendicular to the surface (Holst-Jensen and Schumacher 1994), meaning that this species meets the basic morphological criteria of *Sclerotinia*. However, the position of *S. tetraspora* within *Sclerotiniaceae* remains uncertain (Holst‐Jensen et al. 1998). Based on their ITS phylogeny, Holst‐Jensen et al. (1998) noted that *S. tetraspora* belongs to *Sclerotinia* sensu lato but cannot be included in *Myriosclerotinia* or *Sclerotinia* sensu stricto. Subsequent studies showed that *Sclerotinia tetraspora* is closely related to *S. borealis* and *S. glacialis* (Lorenzini and Zapparoli 2016; Baturo-Ciesniewska et al. 2017). Given that *S. borealis* and *S. glacialis* are themselves of uncertain placement, these phylogenies do not conclusively demonstrate that *S. tetraspora* is a member of *Sclerotinia*. The taxonomic identity of *S. tetraspora* cannot be resolved until a more robust phylogeny is produced that better supports its placement.

#### Sclerotinia trillii

Taxon classificationFungiHelotialesSclerotiniaceae

Y. Harada & Narumi, Mycoscience 42 (2): 184 (2001) [MB#474651].

C30BF078-3C94-5DA9-9B53-5882348A1935

##### Type.

JAPAN • Tomakomai: Hokkaido, from leaves of *Trillium tschonoskii*, 21 June 1996, Y.Harada (holotype: HIROSAKI UNI-23933).

##### Discussion.

*Sclerotinia trillii* was described from Japan, where it infected *Trillium* species (Narumi et al. 2001). This species was compared with *S. sclerotiorum*, *S. minor*, *S. trifoliorum*, and *S. nivalis* and was considered distinct based on its relatively large sclerotia and apothecia, culture appearance, apothecium color, sclerotial protein banding patterns, and its ability to infect *Trillium* (Narumi et al. 2001). Pathogenicity trials on *Trillium*, however, included only *S. nivalis* vs. *S. sclerotiorum*, which weakens host specificity as a defining trait. Given the wide host ranges of *S. minor* and *S. trifoliorum*, it remains possible that one or both may also be pathogenic on *Trillium* species. Additionally, Narumi et al. (2001) described *S. trillii* as producing microconidia on sclerotia and old mycelia, although the functionality of these structures was not addressed. While microconidia have been observed in the three core *Sclerotinia* species, these do not function as germinating propagules (Kohn 1979). This lack of functional conidia has been accepted as a defining characteristic of *Sclerotinia* (Whetzel 1945; Kohn 1979). Given the uncertainty surrounding several of the characters used to define *S. trillii*, it would be prudent to re-evaluate the taxonomic status of this species in future studies.

#### Sclerotinia verrucispora

Taxon classificationFungiHelotialesSclerotiniaceae

Baral, Z Mykol 55: 125 (1989) [MB#135633].

F9F5A97D-D444-525F-AFF9-560B92290B27

##### Type.

GERMANY • Stuttgart-Plieningen: Hattenbach, at the base of an ash tree (*Fraxinus*), 7 May 1978, J.Pernpeintner (holotype: HB 2833, isotypes: CUP 61714 & JTP 4480).

##### Discussion.

This species was described from sclerotia found in soil near the base of an ash tree, but its host remains unknown (Baral 1989). *Sclerotinia verrucispora* meets the basic criteria for a *Sclerotinia* species in that it has definite, tuberoid sclerotia, single-celled, hyaline ascospores, and cells of the apothecial outer excipulum that are globose and oriented perpendicularly to the apothecial surface (Baral 1989). Baral (1989) considered this a distinct species based on the rough ornamentation of the ascospores, a feature not observed in the core species described by Kohn (1979). Additionally, Kohn (1979) described the sclerotial medullae of the core *Sclerotinia* species as “textura oblita with strongly gelatinized walls,” whereas sclerotia of *S. verrucispora* have loose, thin-walled textura intricata medullae that are “intercellular with crystals” and completely free from gel (Baral 1989). The species is mentioned only briefly in the publication describing *S. glacialis* (Graf and Schumacher 1995), and to our knowledge this is the only subsequent published use of the name *S. verrucispora*. DNA sequence data will be required to confirm the taxonomic placement of this species.

### Summary of the status of current *Sclerotinia* species

Various characters have been used to describe the 13 *Sclerotinia* species introduced since Kohn (1979). Given that the last revision of the genus recognised only three species (Kohn, 1979), it is logical that putative new species should be compared primarily with these core species. However, many publications describing new species make comparisons with taxa whose own taxonomic status is uncertain (Gamundi and Spinedi 1987; Graf and Schumacher 1995; Wang et al. 1995; Narumi et al. 2001). As a result, clear conclusions regarding species boundaries are difficult to draw, and a unified approach to characterising species is needed to determine whether they belong in *Sclerotinia*. Fungal taxonomy has increasingly moved toward species delineation based on DNA sequence data (Taylor et al. 2000; Aime et al. 2021; Visagie et al. 2025). However, sequence data were used to support only two of the 13 *Sclerotinia* species described after Kohn (1979), and only three additional species have been included in phylogenetic analyses in subsequent publications. Future efforts to resolve the placement of newly described *Sclerotinia* species will therefore need to incorporate DNA sequence data, ideally obtained from type material. This may prove challenging, as sequences from ex-type material are currently available only for *S. nivalis* (EU330400.1) and *S. cirsii-spinosissimi* (GQ848548.1), the latter of which may belong in *Botrytis* (see the *S. cirsii-spinosissimi* account above).

Including the 13 species described since Kohn (1979), together with the three species she accepted, brings the total number of possible *Sclerotinia* species to 16 (Table 3). Based on the information summarised above, *S. asari*, *S. ginseng*, *S. glacialis*, *S. nivalis*, *S. subarctica*, *S. tetraspora*, *S. trillii*, and *S. verrucispora* meet the morphological criteria for *Sclerotinia*, and some evidence has been presented to support their recognition as distinct species, although further investigation is required in several cases. *S. himalayensis* could not be evaluated because the protologue was unavailable. The identities of *S. antarctica* and *S. atrostipitata* also remain doubtful. The former meets the basic morphological criteria of *Sclerotinia*, whereas only some of these criteria can be assessed for the latter, and for neither species is a clear justification provided for species-level distinction. *Sclerotinia cirsii-spinosissimi* has been transferred to *Botrytis* as *Botrytis cirsii-spinosissimi* (Baral and Quijada 2020). Finally, *S. pseudoplatani* is unlikely to belong in *Sclerotinia*, as there is no evidence that it meets the morphological criteria of the genus, and it was originally noted as a close relative of *Ciborinia candolleana*. Excluding *S. cirsii-spinosissimi* and *S. pseudoplatani* therefore reduces the total number of possible *Sclerotinia* species to 14.

**Table 3.** Overview of current *Sclerotinia* species. List of all *Sclerotinia* species accepted by Kohn (1979) and all legitimate names described since her monograph.

Taxon Name	MycoBank number	Authors	Year of publication
Accepted *Sclerotinia* species			
*Sclerotinia sclerotiorum*	212553	(Lib.) de Bary	1884
*Sclerotinia minor*	271273	Jagger	1920
*Sclerotinia trifoliorum*	207563	Erikss.	1880
Likely an independent *Sclerotinia* species			
*Sclerotinia asari*	108507	Y. Wu & C.R. Wang	1983
*Sclerotinia ginseng*	414139	C.R. Wang, C.F. Chen & J. Chen	1995
*Sclerotinia glacialis*	363084	F. Graf & T. Schumach.	1995
*Sclerotinia nivalis*	442361	I. Saito	1997
*Sclerotinia subarctica*	586496	L.M. Winton, A.L. Krohn & R.H. Leiner ined.	2007
*Sclerotinia tetraspora*	362729	Holst-Jensen & T. Schumach.	1994
*Sclerotinia trillii*	47465	Y. Harada & Narumi	2001
*Sclerotinia verrucispora*	135633	Baral	1989
Likely *Sclerotinia*, but independence uncertain			
*Sclerotinia antarctica*	130648	Gamundí & Spinedi	1987
*Sclerotinia atrostipitata*	133749	Svrček	1988
Not *Sclerotinia*			
*Sclerotinia pseudoplatani*	323267	Svrček	1979
Possible *Botrytis* species			
*Sclerotinia cirsii-spinosissimi*	516616	Senn-Irlet	2016
Cannot be determined at present			
*Sclerotinia himalayensis*	107281	M.P. Sharma & K.S. Thind	1983

### The future of *Sclerotinia* taxonomy

The landmark monograph by Kohn (1979), published more than 45 years ago, remains foundational for *Sclerotinia* taxonomy, establishing the core species and clarifying many previously ambiguous names. Subsequent descriptions of new species have further expanded the genus, yet they have also revealed the limitations of traditional morphological approaches. The work of Holst-Jensen et al. (1997), Ekanayaka et al. (2019), and Johnston et al. (2019) has demonstrated the value of molecular data in addressing some of these limitations. This development also aligns with a broader trend in mycology—the increasing centrality of phylogenetic approaches for delimiting fungal lineages.

Despite the proven utility of molecular phylogenetics in resolving relationships at the family level (Holst-Jensen et al. 1997), a comprehensive, multigene-based reassessment of the genus *Sclerotinia* remains lacking. This situation closely parallels the former state of *Ceratocystis*, a genus that also encompassed diverse but morphologically similar taxa until multigene phylogenies prompted its reclassification into multiple genera (de Beer et al. 2014, 2017; Mayers et al. 2015). The confusion surrounding *Ceratocystis*—described as a genus in which species were “roughly lumped together” based on morphology alone—aptly reflects the current challenges in *Sclerotinia*. As with *Ceratocystis*, the development of a robust phylogenetic framework for *Sclerotinia* would clarify generic and species boundaries across the group.

Two major limitations currently hinder the application of phylogenetic approaches in *Sclerotinia*: the lack of a reliable DNA barcode with sufficient resolution to distinguish among species and the absence of ex-type cultures with associated molecular data. Although the ITS region is the formal DNA barcode for fungi (Schoch et al. 2012), its limited variability across *Sclerotiniaceae* (Holst‐Jensen et al. 1998) may compromise its effectiveness for species-level resolution in *Sclerotinia*. It is therefore essential to evaluate the utility of ITS and, if necessary, identify alternative loci that may serve as more informative barcoding markers. Compounding this issue is the scarcity of viable type strains. Although Kohn (1979) re-typified *S. sclerotiorum*, *S. minor*, and *S. trifoliorum*, these are non-living fungarium specimens, restricting their use in molecular analyses. The practical challenges associated with recovering DNA from aged, non-living material or destructively sampling type vouchers present significant technical and ethical obstacles. The stabilization of names through epitypification therefore represents a critical step, as it ensures that species are anchored to well-characterised, publicly accessible living cultures and provides a foundation for reliable molecular and phylogenetic studies.

Genomic resources represent a largely untapped opportunity for resolving these taxonomic issues. As of August 2025, more than 160 *Sclerotiniaceae* genomes covering nine genera are publicly available through NCBI, although *Botrytis* is overrepresented, with 92 genome sequences. Although some genomes have been used to investigate the biology and pathogenicity of *S. sclerotiorum* (Hossain et al. 2023), they remain underutilised for taxonomic purposes. Genome-scale data could be readily incorporated into multilocus phylogenies and phylogenomic analyses, accelerating efforts to clarify species boundaries and uncover cryptic diversity.

An updated taxonomic revision of *Sclerotinia* could also address unresolved species placements. Kohn’s work eliminated many invalid names but also highlighted numerous doubtful taxa, several of which remain poorly understood. The ongoing ambiguity surrounding *Sclerotinia borealis* exemplifies how unresolved classifications continue to undermine taxonomic clarity. Although removed from *Myriosclerotinia* based on morphological and later molecular data (Schumacher and Kohn 1985; Holst‐Jensen et al. 1998), the species has yet to be formally reassigned, despite suggestions to consider its placement in *Ciborinia*. The continued use of the name *Sclerotinia borealis* (Mardanov et al. 2014) illustrates the persistent instability within the genus. Similarly, newly described species such as *S. trillii* and *S. glacialis* remain phylogenetically unanchored, further emphasising the need for taxonomic revision grounded in molecular evidence.

Despite its economic importance and long history of study, the taxonomy of *Sclerotinia* remains largely unresolved. However, incorporating molecular data offers a clear path forward. Establishing a robust taxonomic framework supported by validated reference strains and informative DNA barcode markers will allow clarification of the currently tenuous identities of some newly described species, such as *S. trillii* and *S. glacialis*, and will refine species limits within the core taxa. A clearer understanding of the taxonomic relationships among *Sclerotinia* species will also facilitate research into areas such as pathogenicity and population genetics, which may ultimately contribute to improved management of the devastating diseases caused by these pathogens.

## Supplementary Material

XML Treatment for Sclerotinia sclerotiorum

XML Treatment for Sclerotinia minor

XML Treatment for Sclerotinia trifoliorum

XML Treatment for Sclerotinia antarctica

XML Treatment for Sclerotinia asari

XML Treatment for Sclerotinia atrostipitata

XML Treatment for Sclerotinia cirsii-spinosissimi

XML Treatment for Sclerotinia ginseng

XML Treatment for Sclerotinia glacialis

XML Treatment for Sclerotinia himalayensis

XML Treatment for Sclerotinia nivalis

XML Treatment for Sclerotinia pseudoplatani

XML Treatment for Sclerotinia subarctica

XML Treatment for Sclerotinia tetraspora

XML Treatment for Sclerotinia trillii

XML Treatment for Sclerotinia verrucispora

## Additional information

### Use of AI

AI was used to edit this article for clarity and language.

### Adherence to national and international regulations

All the fungal strains used in this study have been legally obtained, respecting the Convention on Biological Diversity (Rio Convention).

### Author ORCIDs

Chanel Thomas https://orcid.org/0000-0002-8241-7111

P. Markus Wilken https://orcid.org/0000-0003-0827-6098

Martin P. A. Coetzee https://orcid.org/0000-0001-7848-4111

Cobus M. Visagie https://orcid.org/0000-0003-2367-5353

### Data availability

All of the data that support the findings of this study are available in the main text or Supplementary Information.

## Supplementary materials

Supplementary material 1 Supplementary information Data type xlsx https://binary.pensoft.net/file/1517864This dataset is made available under the Open Database License (http://opendatacommons.org/licenses/odbl/1.0/). The Open Database License (ODbL) is a license agreement intended to allow users to freely share, modify, and use this Dataset while maintaining this same freedom for others, provided that the original source and author(s) are credited. Chanel Thomas, P. Markus Wilken, Martin P. A. Coetzee, Cobus M. Visagie
